# PDMS-PDMS Micro Channels Filled with Phase-Change Material for Chip Cooling

**DOI:** 10.3390/mi9040165

**Published:** 2018-04-02

**Authors:** Zong Liu, Siyin Qin, Xingwei Chen, Dazhu Chen, Fei Wang

**Affiliations:** 1 Department of Electrical and Electronic Engineering, Southern University of Science and Technology, Shenzhen 518055, China; victorliu612@gmail.com (Z.L.); chenxw@mail.sustc.edu.cn (X.C.); 2 Shenzhen Key Laboratory of Polymer Science and Technology, College of Materials Science and Engineering, Shenzhen University, Shenzhen 518055, China; syqin@email.szu.edu.cn (S.Q.); dzchen@szu.edu.cn (D.C.); 3 State Key Lab of Transducer Technology, Shanghai Institute of Microsystem and Information Technology, Chinese Academy of Sciences, Shanghai 200050, China

**Keywords:** MEMS, polymer, micro-channel device, phase-change material, chip cooling, thermal management, flexible electronics

## Abstract

This paper reports on a chip cooling solution using polydimethylsiloxane (PDMS) based microfluidic devices filled with *n*-Octadecane. A thick SU-8 layer of 150 µm is used as the master mold for patterning PDMS fabrication. With the SU-8 mold, patterns with straight lines at microscale have been fabricated with standard micro-electro-mechanical system (MEMS) technology. Thermal polymer bonding technique is used to bond the PDMS pattern directly to a flat polydimethylsiloxane (PDMS) film which results in the sealed microchannels. *n*-Octadecane as a phase-change material has been successfully filled in the microchannels using a dispensing machine. Infrared thermal image shows a sharp contrast of the temperature distribution between the chip with *n*-Octadecane and the empty chip during the same heating process. This result indicates an efficient cooling performance of the microchannel device with phase-change material. A thermal stimulation test demonstrates that a 16 °C-lower temperature difference can be achieved. This microchannel device, benefited from the flexibility of PDMS substrate, shows specific advantages in meeting the need for the heat dissipation of flexible electronics such as flexible displays, electronic skins, and wearable electronics. Latent heat of the phase-change material can keep the temperature of devices relatively lower over a period of time, which shows potential application values on discontinuously active flexible electronic devices.

## 1. Introduction

As more and more components are integrated per unit area, electronic devices are having more complicated functions with better performance. On the other hand, unfortunately, a more severe problem of heat dissipation has become the “hot” topic of microelectronics [1,2,3,4], which also relates to high power consumption per unit area. The overall reliability and lifetime of laptops, smart phones, and lithium batteries have been threatened by the elevated temperature from over-heating. A proper cooling solution is necessary to solve the thermal management challenge for “More than Moore” semiconductor devices. 

New materials with superior thermal conductivity, such as carbon nanotubes, have recently been used to enhance the heat dissipation [5,6,7]. Other materials such as phase-change materials (PCMs) have also been used since they can store or release large quantities of latent heat within a narrow temperature interval during phase change, which have shown great potential in the fields of energy storage and thermal regulation [8,9,10,11].

Micro-channel structures have also been widely researched to cool down the microelectronic chip either with air or filling with super-moist fluids [12,13,14,15]. Youngcheol Joo et al. fabricated metal microchannels through a single-mask process and achieved an economical chip cooling solution with air as the coolant [12]. Kailun Zhang et al. fabricated microchannels filled with super-moist fluids and nanofluids to form a thermosyphon boiling system [14]. In these works, the fabrication of microchannels becomes a crucial part for chip cooling. Different from a traditional heat sink with static fins, Yi, P. et al. proposed dynamic nanofin heat sinks magnetically controlling CrO_2_ nanoparticles onto hot spots, which significantly increased the heat sinking efficiency [16]. 

Droplet-based cooling technologies also provided novel strategies for chip cooling. Electrowetting phenomenon was adapted in chip cooling by moving the droplet along the substrate [17]. Electrical induced oscillation by periodic direct current (DC) pulse or optimized alternating current (AC) pulse of micro-liter sized droplet enhanced with nanoparticles also shows a potential application in hot-spot cooling [18,19]. 

In recent years, flexible electronics appeared in people’s vision and were regarded as a promising technology that may have a revolutionary influence on the future of electronic devices. However, research about a cooling solution aiming at flexible electronics is still not sufficient. Cooling systems designed for chips or other solid electronic devices are usually rigid and not suitable to be applied to flexible devices. Pola T. tested flexible heat sink substrates to achieve a better heat dissipation performance for wearable devices and found that the heat conduction depended on the amount of metal in the tested material [20]. However, the design could not keep a constant temperature. Oshman C. proposed a flat flexible polymer heat pipes that were a highly flexible thermal management solution [21], while the size was not small enough to be applied on fine flexible electronics, such as electronic skins, and wearable electronics. 

PDMS, as a well-studied micro-electro-mechanical system (MEMS) material, can be perfectly integrated with flexible electronic devices because of its flexibility and elasticity. The application of PDMS to embedded electronic components required an improvement in heat exchange. Incorporating synthetic micro-diamond can change the physicochemical properties and enhance the thermal conductivity of PDMS [22]. It has been proven that 10% w/w Al_2_O_3_ nanoparticles in PDMS can significantly increase the thermal conductivity [23]. In this study, we have developed a chip cooling solution using PDMS microchannels filled with PCM. PCMs can absorb or release large amounts of latent heat from the ambient environment through changes in state or structure within a quite narrow temperature scope [24], and have shown great potentials in waste heat utilization [25], energy-storing building [26,27], solar energy storage systems [28], thermal comfort textiles [29], and heat management of electric devices [30,31], etc. Various PCMs, including organic, inorganic, and eutectic ones [32] have been adopted as the latent heat-type functional material, among which, *n*-Octadecane, one of the organic PCMs, was utilized most widely due to its high heat storage density, mild phase-change temperature, and good chemical stability. However, there are some obstacles such as leakage of melted *n*-Octadecane, and volume change during the phase change, which limits its practical use for micro scale devices. To overcome these problems, it was generally microencapsulated with either inorganic materials [10,33] or organic polymers [34,35,36]. In this paper, we reported, for the first time, a novel technique of encapsulating PCMs into MEMS device with parallel straight-lines microchannels for chip cooling application.

**Figure 1:**
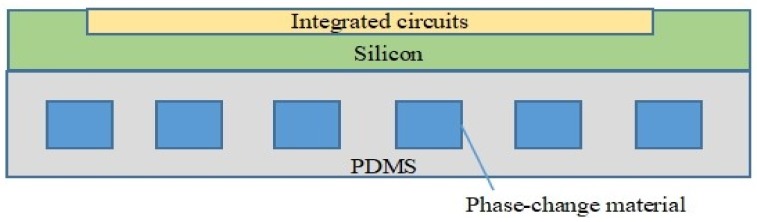
A schematic diagram of the polydimethylsiloxane (PDMS) based microfluidic device filled with phase-change material for chip cooling in integrated circuits.

As shown in Figure 1, PDMS based microchannel device can be fabricated with MEMS processes which are compatible with standard integrated circuits (IC) technology. This design takes the advantages of both the PCM and the microchannel structure, which could help to cool down the over-heated microelectronic chip efficiently. Through a heating test for a prototype device, the PDMS-based microchannel device with PCM can lower the temperature of devices over a period of time, which is efficient for device cooling. Furthermore, this patch-like cooling device is flexible and compact in size, which makes it simple and accessible enough to be combined with flexible electronics. In this way, it is outstanding among other cooling solutions which were generally rigid or too large to apply in flexible electronics. The flexible PDMS-based microchannel device with PCM has demonstrated a promising application for future flexible electronics.

## 2. Materials and Methods

**Figure 2:**
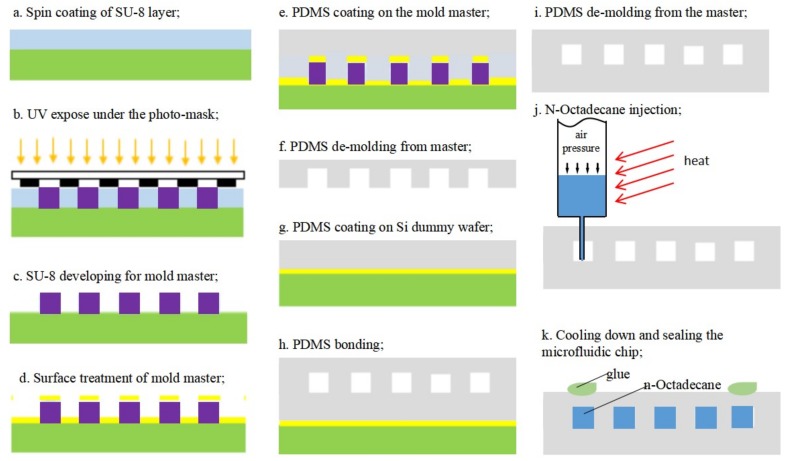
The fabrication process for the microfluidic device, including the master mold fabrication (**a**–**d**); PDMS microchannels (**e**–**i**); and the injection of phase-change material (**j**,**k**).

Figure 2 shows the process flow of the microchannel chip with the *n*-Octadecane phase-change material. The fabrication details are introduced as follow.

### 2.1. Fabrication of the Mold Master

**Figure 3:**
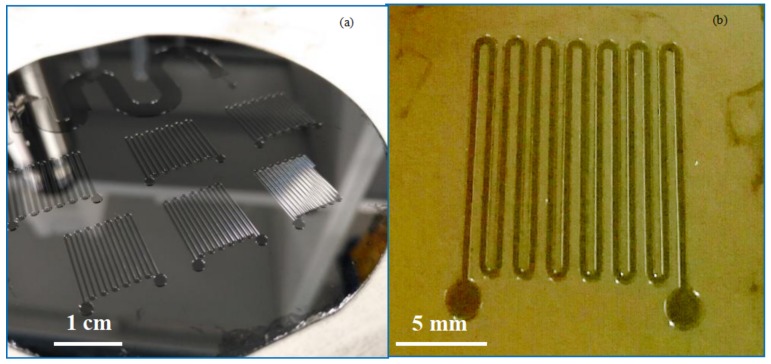
The fabricated SU-8 master mold for the following PDMS casting. (**a**) a few molds on a 4-inch silicon wafer; (**b**) close-up view of one mold for channels with straight lines.

PDMS is a popular material used in the fabrication of microfluidic chips based on injection molding technology. SU-8 is a commonly used epoxy-based negative photoresist, which has been proved as a good mold material for PDMS pattern. The thickness of the SU-8 could be tuned by the spin-rate during coating, and the structure with micro scale dimension could be easily fabricated with optical lithography. Therefore, high aspect ratio up to 20:1 can be achieved for SU-8, which makes it suitable as master mold to fabricate the micro channels [37,38]. A novel modification of conventional microfabrication steps to fabricate large-area, high-aspect-ratio SU-8 molds for PDMS microfluidic devices were shown by Natarajan S. et al. [39]. In 2014, Aaron J Dy et al. introduced two approaches to master mold fabrication using SU-8, which can be applied to many lab-on-a-chip functions [40]. In our study, a thick layer photoresist (SU-8 1075, Gersteltec Sàrl, Pully, Switzerland) was used to obtain a master mold with designed channel geometry of 2-D pattern and a specific depth. On a 4-inch silicon wafer, we first spin-coated the SU-8 1075 resist at 1200 rpm, which gave a layer thickness of 150 µm measured by a step profiler (Dektak-XT-A, Bruker Corporation, Billerica, MA, USA). A standard soft bake was followed at 120 °C for 5 min (Figure 2a). With a photo-mask, the SU-8 layer was exposed to ultraviolet (UV) light (Figure 2b) and developed in propylene glycol monomethyl ether acetate (PGMEA) after a post-bake at 95 °C (Figure 2c). To avoid adhesion problems during the following PDMS de-molding process, the SU-8 master mold was treated with a release agent (release M 4030 from D&D Chemical, Shanghai, China) (Figure 2d). Figure 3 shows the microscopic pictures of the completed mold master with straight line geometry. 

### 2.2. Fabrication of PDMS Microchannels

PDMS was prepared by mixing the prepolymer and curing agent at a weight ratio of 10:1 (Dow Corning SYLGARD184, Midland, MI, USA). The mixture was degassed in a vacuum chamber to remove the air bubbles. Then, the PDMS was poured onto the SU-8 master mold and spin-coated at 400 rpm for 30 s, which gave a layer thickness of about 320 µm measured in the microscopic photo of the cross-section as shown in Figure 8 in the following pages (Figure 2e). After a thermal curing process at 70 °C for 2 h, the PDMS film was peeled off the SU-8 mold as shown in Figure 2f. On another silicon dummy wafer, PDMS film was spin-coated at 500 rpm for 30 s after surface treatment with the release agent mentioned above. As shown in Figure 2g, this process gives a flat PDMS film with a thickness of about 180 µm measured from the cross-section in Figure 8. The PDMS film with patterns will be bonded to the flat PDMS film (Figure 2h). 

**Figure 4:**
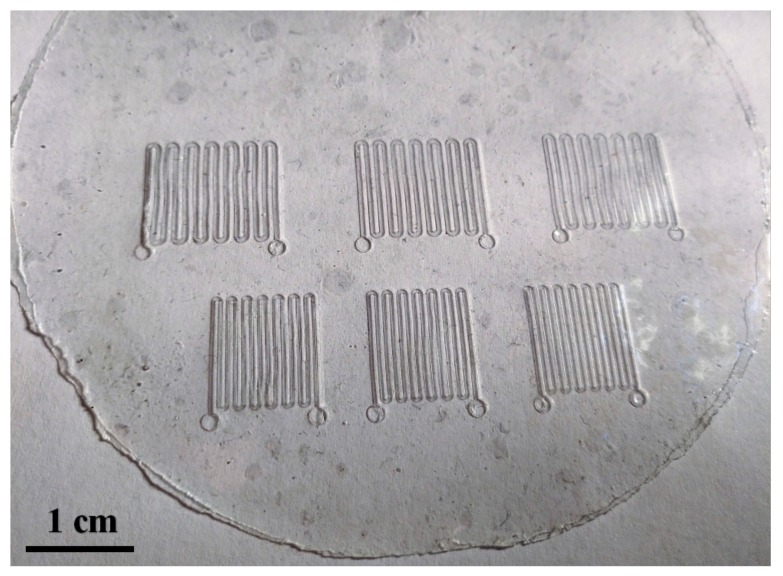
Overall, six microfluidic chips are fabricated simultaneously with PDMS-PDMS bonded film.

Afterward, a thermal bonding technique was performed using a custom-made setup at 120 °C for 12 h with a contact pressure of 3.4 kPa. It has been reported previously that uncured PDMS adhesive bonding would result in better bonding strength for PDMS-PDMS bonding than treated by O_2_ plasma or corona discharge [41], although O_2_ plasma treatment could enhance the bonding strength for PDMS-glass bonding [42,43]. Therefore, no surface treatment was applied in our experiment for the PDMS-PDMS adhesive bonding. The bonded films were peeled off from the silicon dummy wafer after the thermal bonding (Figure 2i). Figure 4 shows the microchannel chips in 4-inch wafer scale. 

### 2.3. Injection with Phase-Change Material

**Figure 5:**
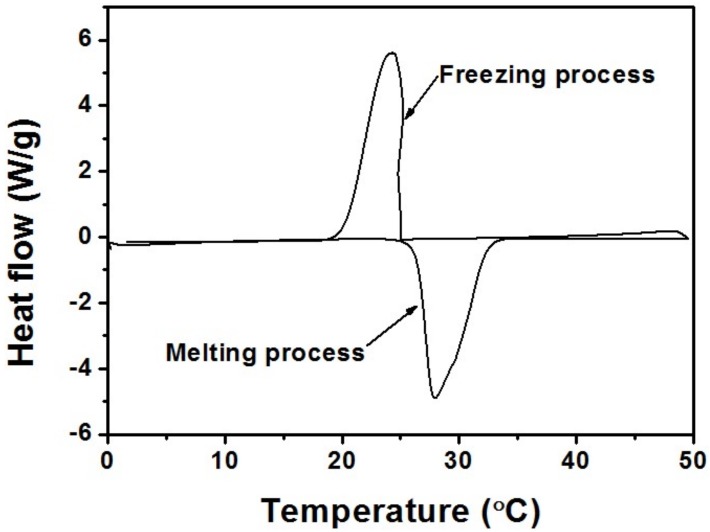
The differential scanning calorimeter (DSC) curve of *n*-Octadecane.

**Figure 6:**
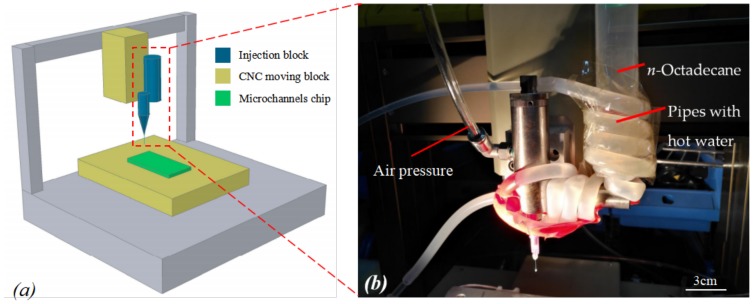
(**a**) The schematic diagram of phase-change material (PCM) injection system using a dispensing machine; (**b**) Close-up view of the injection needle and the container for PCM.

*n*-Octadecane (CH_3_(CH_2_)_16_CH_3_, AR, 98%, purchased from Aladdin, Georgetown, TX, USA) with the density of 0.78 g/cm^3^, thermal conductivity of 0.151 W/m·K and heat capacity of 2.16 J/g·K, is a classification of phase-change material with high melting enthalpy and mild phase-change temperature. When melted, it can be injected into the microchannels. Thermal properties of *n*-Octadecane were determined using a Q200 differential scanning calorimeter (DSC) (TA Instruments, New Castle, DE, USA) at a temperature range from 0 to 50 °C at the heating/cooling rate of 5 °C/min. From the DSC curve presented in Figure 5, the phase-change temperature ranges of *n*-Octadecane are approximately 24.0–34.1 °C for the melting process and 18.1–25.1 °C for the solidification process. The peak melting temperature and latent heat are measured to be 27.9 °C and 216.6 J/g for the fusion process and 24.3 °C and 215.8 J/g for the crystallization process, respectively. As shown in Figure 6a, a dispensing machine was used in order to precisely control the injection position for this specific micro-scale channel. To melt the *n*-Octadecane material, we have twisted a pipe of soft rubber with hot water flowing around the container for *n*-Octadecane, which would keep the temperature of the container higher than the melting point of *n*-Octadecane, as shown in Figure 6b. Meanwhile, the pinhead of the injection system was heated by a lamp to avoid any block from the solidification of *n*-Octadecane. A piezoelectric stage was used to control the position of the needle precisely injecting to the middle of the two PDMS films at the inlet of the microfluidic chip. Then an air pressure was applied to the container to push the liquid-phased *n*-Octadecane into the microchannels (Figure 2j). A hole was poked at the outlet end of the microfluidic chip so that the air could be released when the liquid was injected into the channel. 

**Figure 7:**
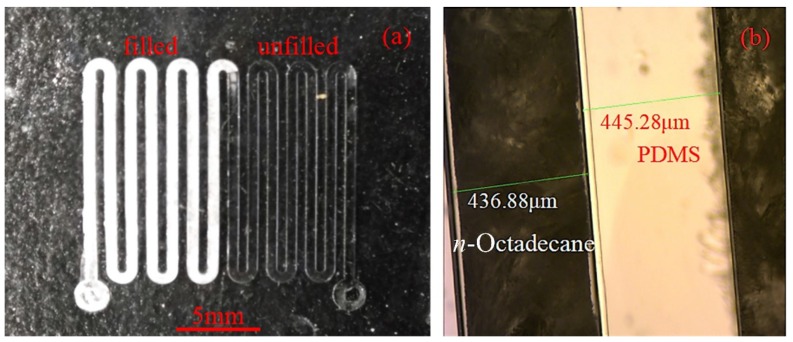
(**a**) The microchannel is partly filled with *n*-Octadecane which could solidify at room temperature; (**b**) the *n*-Octadecane turns to dark under microscopy due to the disorder of crystal grains.

Figure 7a shows a microchannel which has been partly filled with the PCM. A better demonstration can be seen with the videos in the Supplementary Materials, where carbon-ink was manually injected into a microchannel and PCM was injected into another microchannel using the dispensing machine, respectively. When the PDMS microchannel was filled with the liquid *n*-Octadecane, the injection needle was quickly lifted by the stage controller. Finally, the microfluidic chip filled with *n*-Octadecane was quickly cooled down on an ice pack so that the *n*-Octadecane could solidify in the channel. The inlet and outlet openings were then sealed as shown in Figure 2k. Figure 7b shows the microscopic image of the solidified *n*-Octadecane (dark part) in the transparent PDMS chip.

**Figure 8:**
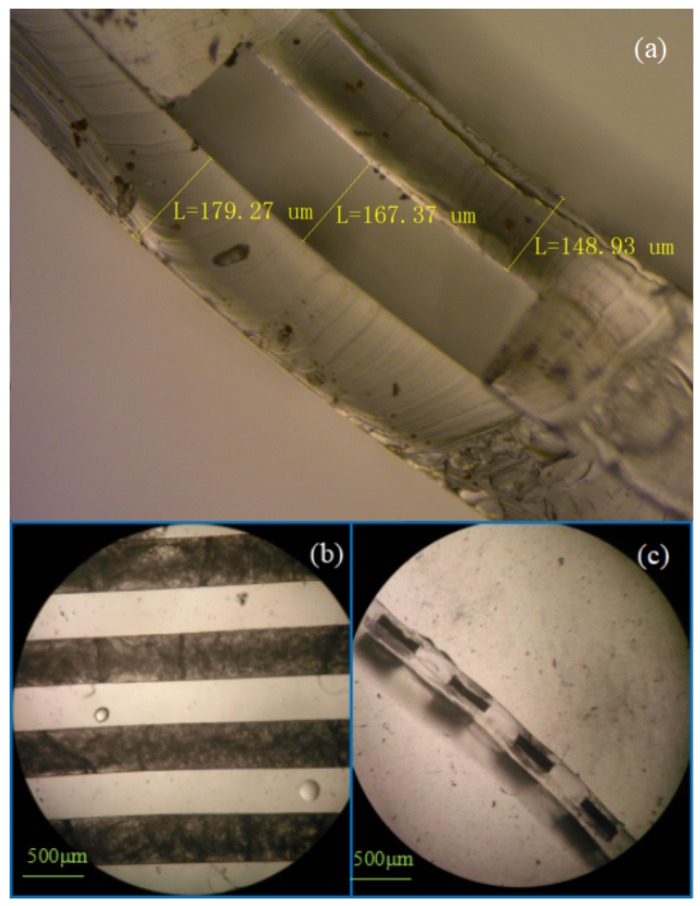
The PDMS-PDMS microchannels (**a**) before PCM filling; (**b**,**c**) after PCM filling.

Figure 8 provides more fabrication details of the device. A cross-sectional view of the microfluidic channel is shown in Figure 8a, where the two PDMS layers are well bonded together. Figure 8b shows the top view of the filled chip while the cross-section is shown in Figure 8c. The *n*-Octadecane material appears dark under the microscope due to the disorder of the crystal grains after solidification.

## 3. Results and Evaluation of the Cooling Performance

**Figure 9:**
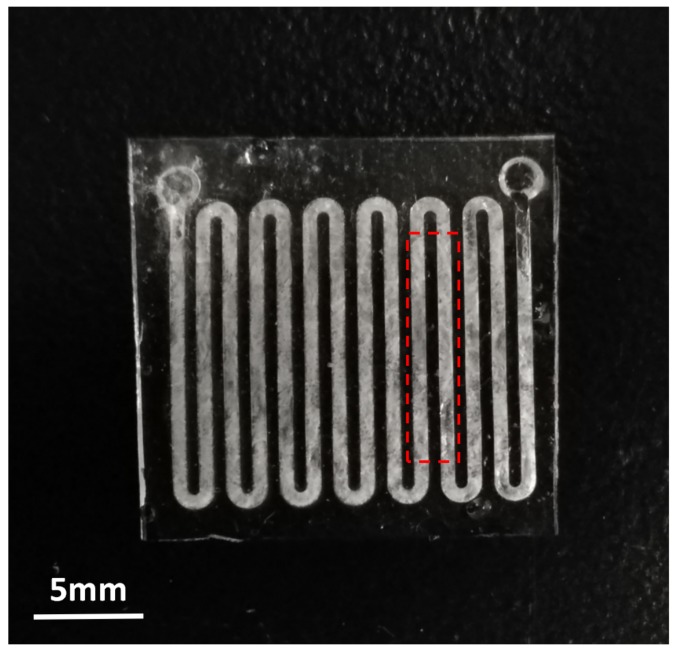
A PDMS-based microchannels device filled with *n*-Octadecane where the wireframe shows the area being cut off for DSC testing.

The energy-storage behaviors of *n*-Octadecane and PDMS microchannels with or without PCM were analyzed using a TA Q200 differential scanning calorimeter (DSC) within the temperature range of 0 to 50 °C at a heating/cooling rate of 5 °C/min under the protection of nitrogen. A sample piece of about 8 mg (the instrument cannot take too much or too little sample for accurate measurement) was cut off from the PDMS microchannels device filled with *n*-Octadecane, which is applicable for the DSC instrument. The sample with a rectangular shape of 11.1 mm × 2.5 mm, including two parallel micro-channels, was tailored for testing as shown in Figure 9.

**Figure 10:**
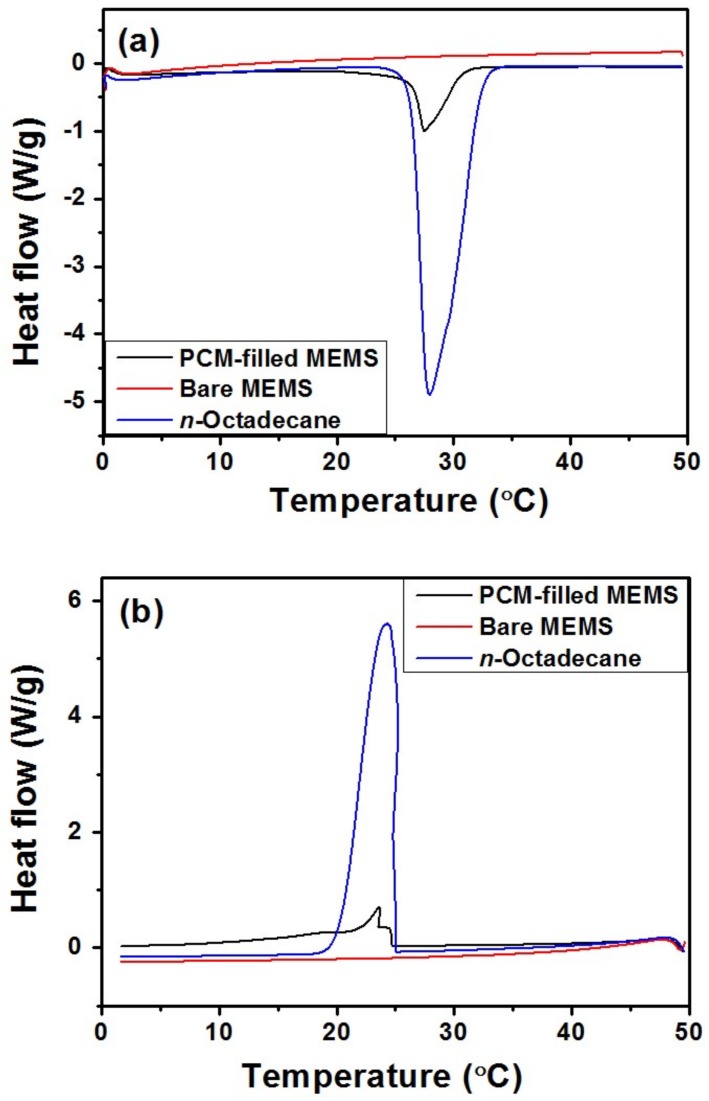
DSC curves of *n*-Octadecane, bare MEMS, and the *n*-Octadecane-filled MEMS during the (**a**) melting and (**b**) freezing process.

Figure 10 shows the DSC curves of *n*-Octadecane and the MEMS cooling device with and without PCM, using the same test parameters as mentioned above. The bare MEMS channel has no endo-/exo-thermic peaks, which means that the thermal effects of the composite MEMS are only attributed to the incorporation of the *n*-Octadecane. During the heating/cooling process, the *n*-Octadecane encapsulated within PDMS microchannels shows a slight delay in both crystallization and melting were observed, because of the low thermal conductivity of PDMS. The stored or released latent heats of the MEMS filled with PCM reach to 34.74 J/g and 38.11 J/g, indicating good ability for thermal energy storage or temperature regulation. 

**Figure 11:**
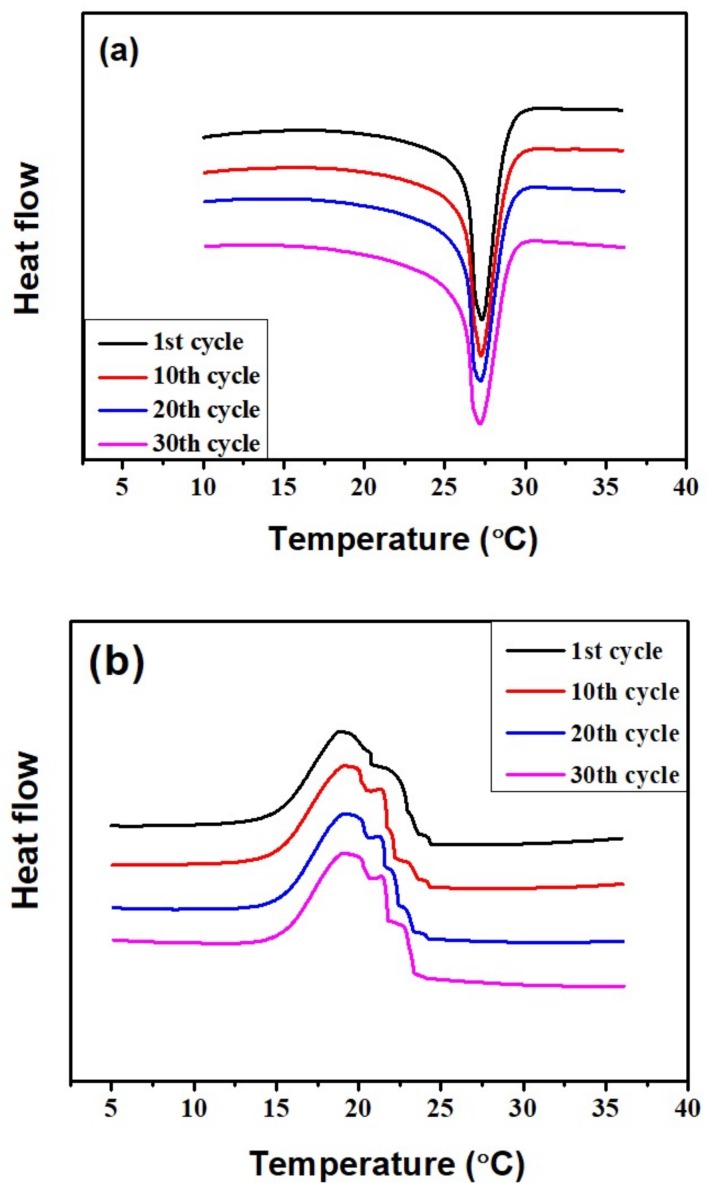
DSC thermograms of PCM-filled MEMS after undergoing 1, 10, 20, and 30 heating/cooling cycles during the (**a**) melting and (**b**) freezing process.

Figure 11 shows the DSC curves of PCM-filled MEMS after 1, 10, 20, and 30 heating/cooling cycles. The PCM-filled MEMS from the first cycle to the 10th, 20th, and 30th cycles shows good coincident melting and freezing peaks, which means the melting and crystallizing temperatures are very close and there is almost no change of the latent heats in the PCM-filled MEMS after experiencing 30 heating/cooling cycles. The PCM-filled MEMS has demonstrated a good thermal cycling reliability. 

**Figure 12:**
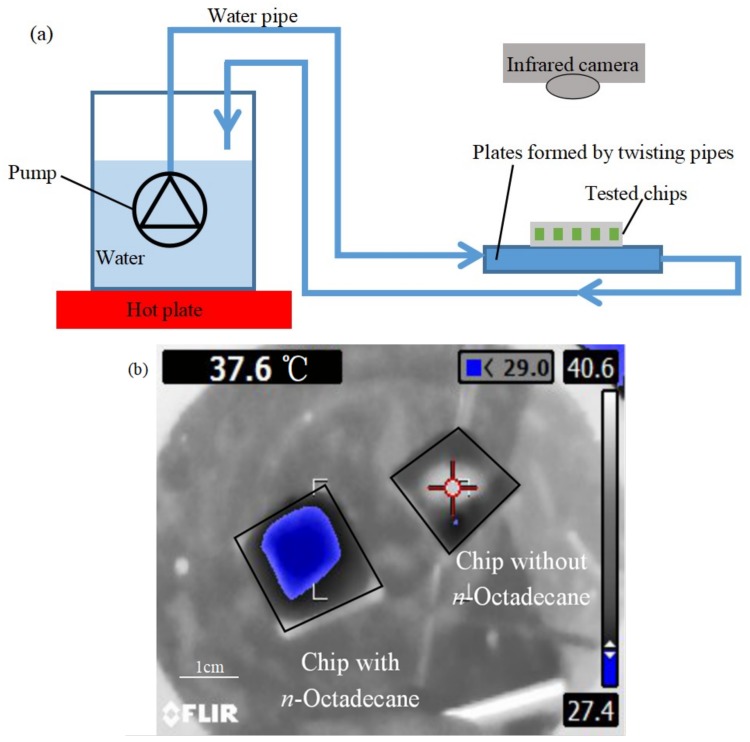
(**a**) A schematic diagram of the test system with a hot water circulation system to control the substrate temperature and an infrared camera (FLUKE Ti110) to measure the chip temperature distribution; (**b**) The comparison between a device filled with *n*-Octadecane and an empty device (the blue zone shows temperature of 27.4 °C for the device with *n*-Octadecane).

To characterize the chip cooling performance of our device, we have designed a test setup with a hot water circulation system to control the substrate temperature and an infrared (IR) camera (FLUKE Ti110, Everett, WA, USA) to measure the chip temperature distribution, as shown in Figure 12a. For comparison, two pieces of devices in the same size with the same structure were tested, one of which was filled with *n*-Octadecane while the other one was empty. The two devices were put on a silicon substrate wafer which was gradually heated up by the hot water circulation system. 

When the temperature of the substrate was increased from room temperature (27 °C) to 40 °C, the infrared thermal image of the two devices were taken, as shown in Figure 12b. From the image, we can see that the microchannel of with *n*-Octadecane has significant cooling effect on the device as the temperature of the device is kept at 27.4 °C; while without *n*-Octadecane, the temperature of the other device is increased up to 40 °C together with the substrate. This demonstration proves that the PDMS-PDMS microchannel filled with *n*-Octadecane can be useful for the chip cooling application.

It should be noted that the microfluidic device should have higher thermal absorption capability when the volume ratio of *n*-Octadecane to the whole device increases. Therefore, decreasing the thickness of the PDMS is an approach for better thermal management performance. A more detailed study of the geometry effect on the cooling efficiency is undertaken and will be shown soon.

**Figure 13:**
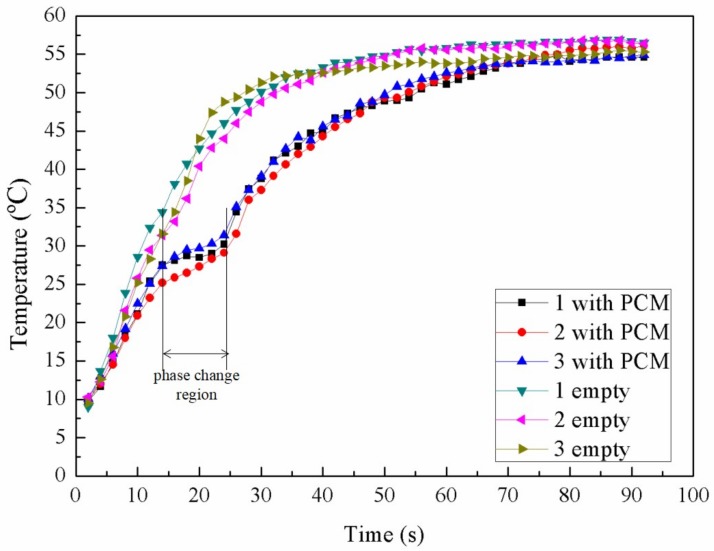
The temperature variation of the three empty chips and three chips with PCM over time.

To study the cooling effect quantitatively, a water bath heating system was set up. Three chips filled with PCM and three empty chips were examined by heating process separately. They were attached to rectangular silicon pieces of the same size which were heated up simultaneously. These chips were heated from 10 °C by a heat source of constant 65 °C when the room temperature was 24.5 °C. During the heating process, the chips can continuously absorb heat from the source until a final thermal equilibrium was achieved. Figure 13 shows the curves of the temperature variation over time. Either with or without PCM, the three chips for each type exhibit the same trend for the measured curve, which proves excellent repeatability of the devices during the test. For empty chips, the smooth curves show a continuous increase in the temperature which saturated at approximately 55 °C when reaching the equilibrium. On the other hand, a comparatively flat stage was observed for the chips with PCM, which indicates that PCM in the microchannel undergoes a phase change period and a large amount of heat is absorbed with little temperature increase. The large gap between the temperature curves of the two types of devices has clearly demonstrated the cooling effect of PCM. This phase change period plays an important role in the cooling performance.

**Figure 14:**
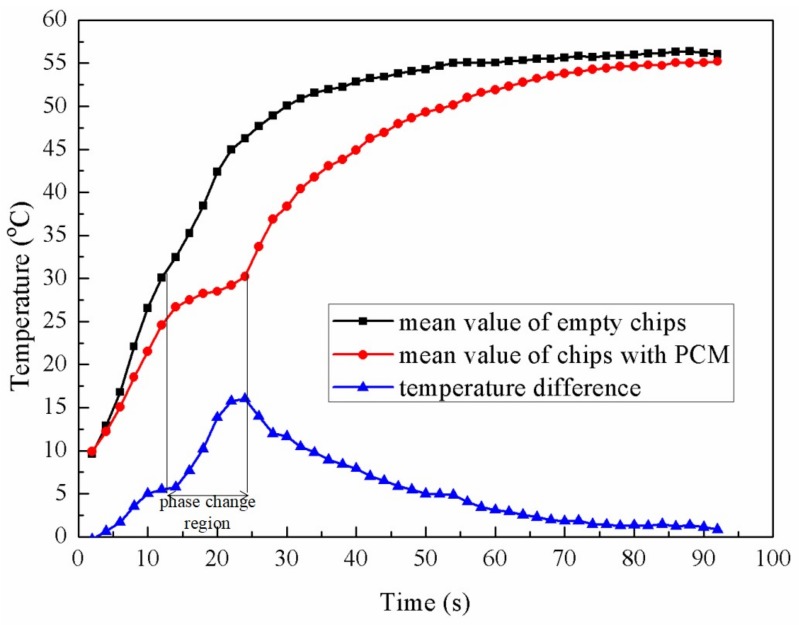
Mean value of the temperature of three empty chips, mean value of the temperature of three chips with PCM, and the temperature difference during the heating test.

In order to take a deeper investigation on the temperature changing over time especially the phase change period, Figure 14 shows the mean value of three empty chips and three chips filled with *n*-Octadecane, respectively. From the figure, there is a relatively flat section of the curve around 26–30 °C for the chips with PCM. During this period, the temperature difference between two types of chips increases rapidly, reaching the maximum value of 16 °C at the end of phase change period when the mean temperature of the chips with PCM is 29 °C while the chips without PCM is about 46 °C. If we define a ratio *η* as the temperature difference between the two chips divided by the mean temperature of the chip without PCM, this ratio of *η* goes up to 35.1% at this point, which shows an efficient cooling effect. Before and after the phase changing region, the mean temperature of chips with PCM is cooled to 25 °C and 35 °C, with *η* of 18.3% and 25.1%, respectively, which also demonstrates a good function of this cooling strategy. Both empty chips and chips filled with PCM reached a same equilibrium temperature in the end according to the same constant thermal source. 

## 4. Conclusions

In this paper, we have fabricated PDMS based microfluidic chips filled with phase-change material for chip cooling applications. The SU-8 photoresist is used as the master mold for PDMS patterning, and PDMS-PDMS adhesive bonding is applied to achieve sealed channel structure with various patterns at microscale using standard MEMS technology. The channel is filled with *n*-Octadecane as a phase change material which can absorb heat efficiently, which prevents or slows down the temperature rising from the heating source. A prototype device shows that the microfluidic chip with *n*-Octadecane can be kept at room temperature while the other chip without *n*-Octadecane is heated up to 40 °C during the same period. The curves of the variation of temperature over time show a clear thermal property that the temperature can be kept constantly to some extent. A temperature reduction around 16 °C can be achieved at the end of the phase change period of *n*-Octadecane. Furthermore, heating and cooling experiments with different PDMS microchannel devices demonstrate the repeatability of the method and the durability of the PCM. Different from other stiff microfluidic cooling devices designed for macro devices, the flexible PDMS sheet and miniaturized microchannels with PCM make it compatible with the plastic substrates of flexible electronics. This chip cooling solution takes the advantages of both microchannels cooling and phase-change material, which shows a promising application in solving the thermal management issue for future flexible electronics. 

## Supplementary Materials

The following are available online at http://www.mdpi.com/2072-666X/9/4/165/s1, Video S1: Ink.avi; Video S2: pcm.avi; Video S3: heating.avi.
